# Emergency Department: Analysis of Patient Flow and Length of Stay Variations

**DOI:** 10.1093/eurpub/ckac131.302

**Published:** 2022-10-25

**Authors:** E Fanti, M De Marco, C Lorenzini, B Porchia, M Dominijanni, R Gusinu, G Messina

**Affiliations:** 1 Post Graduate School of Public Health, University of Siena, Siena, Italy; 2 Hygiene and Epidemiology Unit, University Hospital of Siena, Siena, Italy; 3 University Hospital of Siena, Siena, Italy; 4 Department of Molecular and Developmental Medicine, University of Siena, Siena, Italy

## Abstract

**Background:**

Crowding in Emergency Departments(ED)is a severe public health issue.Length of stay(LOS)is not a direct measure of crowding,but it is an essential indicator for monitoring emergency care quality.LOS in ED can be associated with delays in treatment,decreased patient satisfaction and adverse outcomes.The aim of this study is to analyze ED LOS in the Teaching Hospital of Siena for further strategies.

**Methods:**

A retrospective observational study was conducted between January 1,2019, and December 31,2021.To manage admissions and discharges, all patients’ data admitted to ED of the University Hospital of Siena were accessed by Aurora,the IT system.In addition,a descriptive analysis was performed, collecting the following variables:sex,age,arrival mode,ED visit reasons,triage code,discharge mode,hospital admission area and LOS(cut-off>8hours).The analysis was carried out using STATA 17:variables were analyzed with ANOVA test.

**Results:**

Our sample consisted of 152.393 patients (F49.47% M50.53%),and the average age was 50.51(SD ± 26.07).During the years total ED visits decreased:65.426(2019);40.318(2020); 46.649(2021),and there was a significant increase (p < 0.001) of patients with LOS>8 hours:13.96%(2019); 21.51%(2020); 23.10%(2021).In the years 2019,2020 and 2021, admissions of patients with LOS>8 hours were respectively: 25.92%; 43.95% and 37.09%, with the following percentage in medical areas:69.96% in 2019;70.51% in 2020;64.55% in 2021.A progressive increase of admissions in COVID area resulted since 2020(2.23%-2020;6.07%-2021).

**Conclusions:**

The spread of COVID-19 and the containment measures,such as lockdown,caused a significant decrease in ED access.The increase LOS>8h could be primarily due to the time needed to perform laboratory investigations for the search for SARS-CoV-2 but also to the overflow of SARS-CoV-2-infected patients rapidly saturating the ED boxes and hospital bed capacity,with the need sometimes to dedicate other medical areas to manage COVID patients.

**Key messages:**

• ED-LOS is a proxy indicator to monitor emergency care quality.

• Further investigations should be performed to analyze the leading causes of ED LOS increase during the pandemic period.

